# Non-pharmacological interventions for depressive disorder in patients after traumatic brain injury: A protocol for a systematic review and network meta-analysis

**DOI:** 10.1097/MD.0000000000022375

**Published:** 2020-09-25

**Authors:** Mingmin Xu, Yu Guo, Yulong Wei, Lu Wang, Xiumei Feng, Yue Chen, Jian Yan

**Affiliations:** a School of Acupuncture-Moxibustion and Tuina/The Third Affiliated Hospital, Chengdu University of Traditional Chinese Medicine, Chengdu; b Teaching and Research Section of Acupuncture; c Formula-Pattern Research Center, School of Traditional Chinese Medicine, Jinan University, Guangzhou; d School of Acupuncture-Moxibustion and Tuina, Beijing University of Chinese Medicine, Beijing, China.

**Keywords:** depressive disorder after traumatic brain injury, network meta-analysis, non-pharmacological interventions, protocol, systematic review

## Abstract

**Background:**

Depressive disorder has gradually become one of the most commonly reported disabling psychiatric complication that occurs after traumatic brain injury (TBI). Currently classical antidepressant medications may not have the same effectiveness in patients with TBI as in patients without TBI. Non-pharmacological interventions have been considered to be effective for managing depressive symptoms or treating depressive disorder. But to date the comparative effectiveness of various types of non-pharmacological interventions has been synthesized in few studies, the evidence remains inconclusive. Thus, the purpose of this systematic review and network meta-analyses is to summarize high-quality evidence and identify the most effective non-pharmacological intervention when applied to treat the depressive disorder in patients after TBI.

**Methods:**

The comprehensive literature search in electronic database including PubMed, Ovid Medline, Cochrane Library, Web of Science database, Embase Database, China National Knowledge Infrastructure (CNKI), and Wanfang Data Chinese database from inception to the search date. Only high-quality randomized controlled trials (RCTs) that have used non-pharmacological interventions to treat depressive disorder after TBI will be considered. Two independent reviewers will identify eligible studies, extract and manage data information, and then determine methodical quality of included studies. Overall efficacy will be assessed as primary outcome. Secondary outcomes involved treatment response, remission rate, overall acceptability, tolerability of treatment, social functioning, occurrence of adverse events, and suicide-related outcome. Cochrane risk of bias assessment tool will be adopted to assess the risk of bias. Study heterogeneity will be measured by the *I*^2^ statistic. Traditional pairwise meta-analyses will be performed using STATA, while WinBUGS with GeMTC package of R software will be used to carry out network meta-analysis.

**Results:**

This systematic review will examine the relative efficacy, effectiveness, safety, tolerability and acceptability of non-pharmacological interventions, and then to identify the most effective non-pharmacological intervention for depressive disorder after TBI.

**Expected conclusion:**

Our work could be used to give clinical recommendations for practice guideline developers, psychiatrist, neurologist, policymakers, researchers as well as individual with depressive disorder after TBI, and will also identify gaps in knowledge that could be the subject of future research.

**Ethics and dissemination:**

Neither ethics approval nor patient informed consent is necessary since this protocol was designed based on the existing literature. The results will be disseminated electronically or in print through publications in peer-reviewed scientific journal.

**INPLASY registration:**

INPLASY202080022.

## 1 Introduction

Depressive disorder, as a common mental state in which a person feels at least 1 episode of either depressed mood or loss of interest and pleasure in usual activities or both consistently for at least a 2-week period.^[1,2]^ Traumatic brain injury (TBI) is defined as an alteration in brain function, or other evidence of brain pathology.^[3]^ The commonly damage to the brain occurs because of external forces.^[3,4]^ TBI affects people of all ages, races, and sexes, and it is becoming increasingly prevalent, as approximately 10 million of deaths and/or hospitalizations annually are directly related to TBI worldwide.^[5]^ Annually in the United States, the incidence of TBI is approximately 1.7 million cases, considering for 50,000 deaths.^[4]^ Furthermore, as the prevalence of TBI continues to increase, each year in the United States, the economic costs were estimated at about $76.5 billion on TBI treatment, rehabilitation, and lost productivity caused by the injury.^[6]^ It is noteworthy that experiencing TBI has been identified as a risk factor for the development of depressive disorder.^[2]^ Individuals with TBI are almost 10 times more likely than the general population to experience a depressive episode during their first year of recovery (53% compared with 6% per 12 months in the general population).^[7,8]^ Depressive disorder after TBI can have severe consequences for rehabilitation outcomes, as well as impact other domains such as cognitive functioning, interpersonal, occupational, social functioning, activities of daily living, and overall quality of life.^[2,7–10]^ What's more, it also represents a significant risk factor for mortality through suicide.^[11,12]^ There is no doubt that effective treatments for depressive disorder after TBI must be developed, tested, and disseminated. It is common knowledge that treatment for depressive disorder in patients with TBI is complicated. Today there is a paucity of evidence-based studies to guide the treatment of depressive disorder after TBI.^[2,9,13]^ Due to the multifactorial biological and psychosocial contributors to depressive disorder after TBI, classical antidepressant medications such as selective serotonin reuptake inhibitors (SSRIs) and tricyclic antidepressants may not have the same effectiveness and tolerability in patients with TBI as in patients without neurological insult.^[2,9,13]^ In addition, some studies suggested that more unwanted adverse events happen in patients receiving these pharmacotherapies.^[14–17]^

Due to the chronicity of depressive symptoms and intolerability to pharmacological treatments, patients with TBI are inclining to choose non-pharmacological interventions as an alternative option or as an add-on treatment. Plenty of randomized controlled trials (RCTs) have been conducted to confirm the effect of non-pharmacological interventions such as psychological interventions, physical interventions, complementary and alternative medicine (CAM) interventions on depressive disorder.^[18–29]^ Recently, non-pharmacological interventions have drawn the attention of investigators. Despite there are a few systematic reviews showed the effectiveness of non-pharmacological and their potential for a lesser tolerability burden in this vulnerable TBI population, unfortunately the reliability of the evidence might be influenced by between-study heterogeneity and other risks of bias.^[7,17,30]^

## 2 Objectives

Based on this, we posed the following questions: are non-pharmacological interventions effective and safe for depressive disorder after TBI? If so, among these non-pharmacological interventions, which is the most comparatively effective, safe, and acceptable intervention to manage depressive symptoms or treat depressive disorder after TBI? To answer these questions, we will perform a systematic review and Bayesian network meta-analysis (NMA) together with traditional pairwise meta-analysis to examine the relative efficacy, effectiveness, safety, tolerability and acceptability of non-pharmacological interventions, and then to identify the most effective non-pharmacological intervention for depressive disorder after TBI.

## 3 Methods and analysis

This systematic review and network meta-analysis has been prospectively registered on the INPLASY website (https://inplasy.com/inplasy-2020-8-0022/) and INPLASY registration number is INPLASY202080022. The systematic review and NMA protocol has been developed the proposed systematic review in accordance with the Preferred Reporting Items for Systematic Reviews and Meta-Analyses Protocols (PRISMA-P) statement guidelines.^[31]^ The study will start on August 30, 2020, and is expected to be completed by November 10, 2020.

### 3.1 Inclusion and exclusion criteria

The eligibility criteria of the studies were established in terms of participant, intervention, comparison, outcome, and study design type (PICOS) approach.

#### 3.1.1 Types of studies

Only high-quality RCTs using non-pharmacological interventions for patients with depressive disorder after TBI will be considered, without any date of dissemination or restriction of language. Furthermore, RCTs with crossover trials will be excluded because the washout periods of non-pharmacological intervention may bring bias to outcome assessments. As it is difficult to use a single-blind or double-blind design for patients in trials of non-pharmacological intervention alone or the combination of active treatments. We will only include studies in which raters or outcome assessors were blinded. Other study designs such as non-randomized clinical trials, quasi-RCTs, retrospective studies, review studies, animal mechanism experiments, case reports, uncontrolled trials, laboratory studies, and cohort studies will be excluded.

#### 3.1.2 Types of participants

We will include studies that enrolled patients who had a disease history of TBI as well as were confirmedly primary diagnosed with depressive disorder, or had clinically significant depressive symptoms, based on at least one of the standardized international or domestic authorized diagnostic criteria or guidelines for clinical research such as Feighner criteria, Research Diagnostic Criteria, Diagnostic and Statistical Manual of Mental Disorders 3rd edition (DSM-III), 3rd revised edition (DSM-III-R), 4th edition (DSM-IV), 5th edition (DSM-5), and International Classification of Diseases10th revision (ICD-10), etc.^[32–34]^ We will not apply restrictions with regard to any information about age, sex, race, education status, nationality, economic status, severity, and duration of disease, etc. A concurrent secondary diagnosis of another psychiatric disorder after TBI will not be considered as an exclusion criterion, but studies in which all patients have a concurrent primary diagnosis of another Axis I or II disorder will be excluded. In addition, the participants with TBI suffering from bipolar disorder, treatment resistant depressive disorder, subthreshold depressive disorder, seasonal affective disorders, peripartum depressive disorder, depressive disorder in dementia, or psychotic depression will be also excluded.

#### 3.1.3 Types of interventions

We plan to include any form of non-pharmacological intervention can be used as monotherapy or combined treatments to reduce depressive symptoms or resolve the presence of a diagnosable depressive disorder after TBI. The non-pharmacological interventions might have been psychological, medical, physical, or CAM interventions, such as cognitive behavioral therapy (CBT), meditation, acceptance, and commitment therapy (ACT), electro-convulsive therapy (ECT), repetitive transcranial magnetic stimulation (rTMS), homeopathy, music therapy, traditional Chinese medicine non-pharmacological intervention (e.g., acupuncture, moxibustion, traditional Chinese exercise Qigong, Tuina, cupping), and so forth. Trials comparing the same type of non-pharmacological intervention, but at different numbers of therapeutic sessions, and different treatment conditions (with or without nurses’ involvement) will be considered as the same node in the NMA. To assess the efficacy, effectiveness, safety, tolerability, and acceptability of non-pharmacological interventions, we plan to compare them with each other, and conventional pharmacological interventions, as well as placebo control, including placebo drugs, sham interventions, no intervention, waiting list membership, etc.

#### 3.1.4 Types of primary outcome

Overall efficacy (as a continuous outcome), it refers to mean improvement in depressive symptoms, as measured by overall mean change scores on continuous observer-rated scale (self-rated or assessor-rated) for depressive disorder from baseline to the end of the study duration.

#### 3.1.5 Types of secondary outcomes

1. Treatment response (as dichotomous outcome), defined as 50% or greater reduction from baseline to study end point in the study's primary observer-rated depression scale.
2. Remission rate (as dichotomous outcome), it refers to by the total number of patients who achieved the criteria of remission, defined as being below the threshold in depressive disorder rating score in different across trials.
3. Overall acceptability (as dichotomous outcome), operationalized as the proportion of participants who terminated the study early owing to any cause up to the end of the study duration.
4. Tolerability of treatment (as dichotomous outcome), defined as the proportion of patients who discontinued treatment due to any adverse events during the delivery of the non-pharmacological interventions.
5. Social functioning (as continuous outcome), as measured by overall change scores on any validated global assessment of functioning scales such as Global Assessment of Functioning (GAF) scale or quality of life scales.^[35]^
6. Occurrence of adverse events (as dichotomous outcome), as reported in the include studies.
7. Suicide-related outcome (as continuous outcome), estimated by the reported the number of patients who deliberately self-harmed, attempted, or completed suicide from baseline to study end point. The definition of suicide-related outcome is based on the standardized, validated, and reliable rating scale, such as Columbia Classification Algorithm of Suicide Assessment (C-CASA).^[36]^

### 3.2 Search methods for identification of studies

#### 3.2.1 Electronic searches

The following electronic databases including PubMed, Ovid Medline, Cochrane Library, Web of Science database, Embase Database, China National Knowledge Infrastructure (CNKI), and Wanfang Data Chinese database. To ensure a broad search, titles, abstracts, and keywords will be searched using a combination of Medical Subject Headings (MeSH) words and free-text terms incorporating database-specific controlled vocabularies and text words related to RCTs, non-pharmacological intervention, depressive disorder or depression, TBI or traumatic brain injury, etc. Example of details of the search strategy for PubMed is shown in Table 1.

**Table 1:**
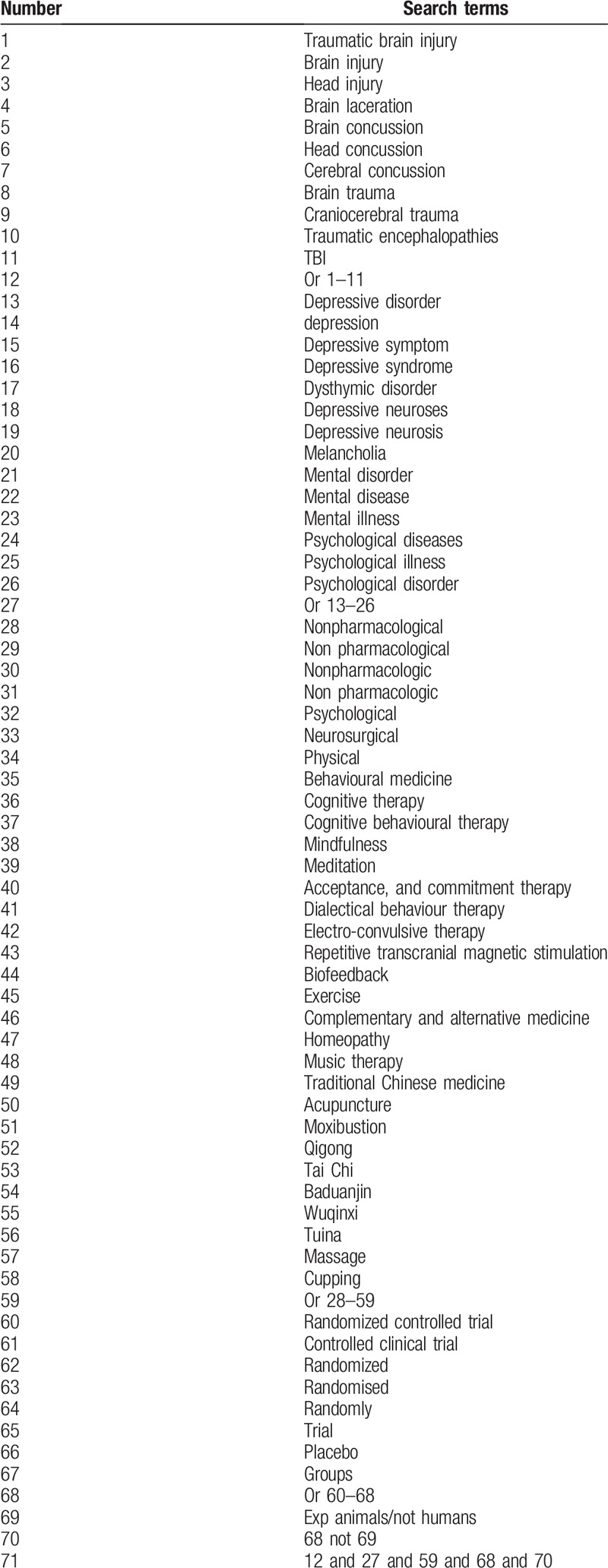
Search strategy for the PubMed database.

#### 3.2.2 Other sources

The reference lists of previously published reviews and selected RCTs will be tracked, and corresponding authors of chosen RCTs will be contacted if it is necessary. A list of medical journals will be hand searched in the university library. Any relevant ongoing or unpublished experimental studies that are relevant to this topic will be gained from the WHO International Clinical Trials Registry Platform (http://www.who.int/trialsearch), meta-Register of Controlled Trials (http://www.controlled-trials.com), United States (US) National Institutes of Health Ongoing Trials Register (http://www.clinicaltrials.gov), and the Chinese Clinical Trial Registry (http://www.chictr.org/cn/). Potential gray literature will be searched in OpenGrey.eu. website. No publication language, publication date, and publication status restrictions will be applied.

### 3.3 Data collection and analysis

#### 3.3.1 Selection of studies

**Figure 1:**
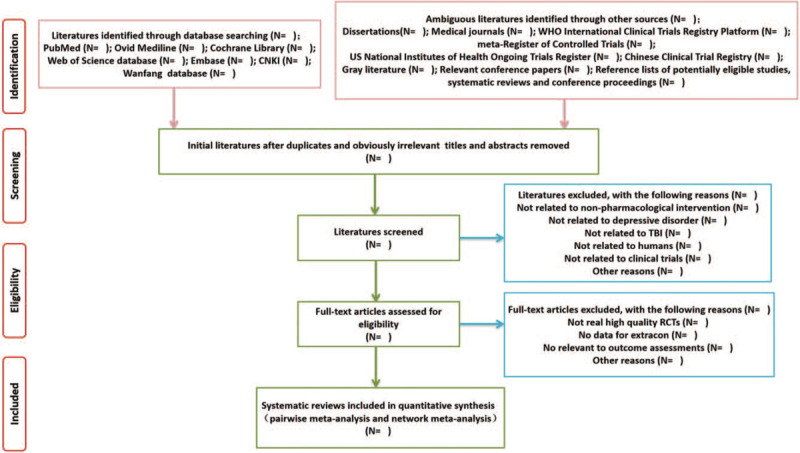
Details of selection procedure of studies retrieved from databases.

Two reviewers (MMX and GY) will independently use EndNote X10 to identify the all potential relevant clinical studies by scanning the titles, abstracts, and keywords. If multiple studies describe the same trial, the study with the most relatively complete data will be used in the analyses. After excluding the duplicated and apparently irrelevant studies, the full-text copies of the remaining studies will be re-evaluation. Disagreements will be resolved by team meeting or consulting a third reviewer (LW). Details of selection procedure of studies retrieved from databases will be shown in Fig. 1.

#### 3.3.2 Data extraction and management

Two independent reviewers (MMX and GY) will extract the necessary data from the included studies according to a standard data extraction: the general information, trial methodological description, study population characteristics, intervention and control characteristics, outcome measures, results, and notes. Then 2 reviewers (MMX and GY) will check the accuracy and consistency of all extracted data. A third reviewer (LW) will be make final decision if the dispute between the 2 reviewers. The following data will be extracted:

1. General information: for example, publication year, the title of study, the first author of article, correspondent author of article, contact information, publication source, country of origin, language of publication, etc.
2. Trial methods: for example, aims and objectives, design of study, inclusion and exclusion criteria, recruitment and sampling methods, number of participants, number of groups, number randomized to each group, method of randomization, method of allocation concealment, details of blinding methods, incomplete report or selecting report, other sources of bias, etc.
3. Participants characteristics: for example, sociodemographic characteristics, mean age, sex, ethnicity, status of depressive disorder after TBI, duration of depressive disorder after TBI, severity of disease history of TBI, research setting, baseline depressive disorder score after TBI, diagnostic criteria used of depressive disorder after TBI if reported, etc.
4. Intervention and control characteristics: for example, type and name of non-pharmacological intervention, type and name of control, program period, intervention frequency, intervention durations, information on intervention providers, who supervised the program (if applicable), etc.
5. Outcomes data: for example, type of outcome, definition of the outcome, time point for assessment, primary, secondary, and safety outcomes as described above, length of follow-up, timing of follow-up measures, and the key findings of the study, including both within-group and between-group differences, etc.
6. Results: for example, mean for both groups, mean difference (MD), SDs/95% CIs/SEs, number of missing patients, statistical methods used and appropriateness of these, efficacy, effectiveness, safety, tolerability and acceptability, and suicide-related outcome, total sample size, etc.
7. Notes: for example, financial support, conflicts interest, ethical approval, important citations, etc.

#### 3.3.3 Quality assessment in included studies

Two reviewers (MMX and YG) will independently evaluate the methodological quality of eligible studies by using the risk of bias (ROB) assessment tool described in the Cochrane Collaboration Handbook.^[37]^ The risk of bias domains including: selection bias (random sequence generation, allocation concealment), performance and detection bias (blinding of therapists and participants), detection bias (blinding of outcome assessors), attrition bias (incomplete outcome data assessment for each outcome, differential dropout), reporting bias (authors of RCTs explained whether reported outcomes were selective or not), other sources of bias (for example, conflicts of interest, follow-up, different characteristics and representativeness of participants, non-intention-to-treat or per-protocol analysis, and so forth). After assessing all the domains, we will evaluate the methodological quality of each study as low, unclear, and high risk of bias. The inter-rater reliability of the 2 reviewers (MMX and YG) assessing the risk of bias will also be calculated. Any discrepancies in judgements of bias will be resolved through discussion with a third reviewer (LW).

#### 3.3.4 Dealing with missing data

We will contact first and/or corresponding author to obtain any missing data. If no one responds, we will try to use the available coefficients to calculate the data. The potential impact of missing data on the results will be tested in sensitivity analysis. The reason for exclusion of the available data will be reported.

#### 3.3.5 Traditional pairwise meta-analysis

If the data are not available for quantitative analysis or information are insufficient, we will summarize the evidence and give a narratively reported regarding the findings of our study. Traditional pairwise meta-analyses will be performed using STATA version 14.0 (Stata Corporation, College Station, TX). The pooled estimates of the weighted mean difference (WMD) or the standard mean difference (SMD) with 95% confidence intervals (CIs) will be calculated for the continuous outcomes, while for dichotomous outcomes, data will be analyzed using the risk ratio (RR) with 95% CIs.^[38]^ Statistical heterogeneity of intervention effects will be assessed by using the *I*^2^ statistic. If the *P* value is ≥.1 and *I*^2^ ≤ 50%, which suggests there is no significant statistical heterogeneity exists, and the Mantel-Haenszel fixed effect model will be employed. If the *P* value is <.1 and *I*^2^ > 50%, and the Mantel-Haenszel random effect model will be used.^[38]^

#### 3.3.6 Bayesian network meta-analyses

We will perform NMA in a Bayesian hierarchical framework^[39]^ using WinBUGS version 1.4.3 (Medical Research Council Biostatistics Unit, Cambridge, UK) with GeMTC package of R version 3.02 software to compare the efficacy, effectiveness, safety, tolerability and acceptability across of selected non-pharmacological interventions for depressive disorder after TBI, and obtain a comprehensive ranking of selected non-pharmacological interventions. We will use the Markov Chain Monte Carlo simulation technique to generate samples. The Brooks-Gelman-Rubin plots method will be adopted to assess model convergence.^[40]^ Convergence will be found to be adequate after running 50,000 samples for each chain. These samples will then be set as the “burn-in” period, and then posterior summaries will be produced based on a further 100,000 subsequent simulations. For primary and secondary outcomes, the ranking probability (best, second-best, third-best, and so on) of each non-pharmacological intervention will be calculated and graphically ranked with rank gram plots, and a treatment hierarchy using the probability of being the best treatment can be obtained. The surface under the cumulative ranking curve (SUCRA) and probability values will be summarized and reported as SUCRA for each non-pharmacological intervention. SUCRA curves will be described with percentages, 100% for the best treatment while 0% for the worst. In this systematic review, both fixed-effect and random-effect models in the Bayesian NMA will be considered based on the results of the deviance information criterion.^[40]^

##### 3.3.6.1 Assessment of heterogeneity

In NMA, we will assume a common estimate for the heterogeneity variance across the different comparisons, and then we will estimate the heterogeneity variance and judge its magnitude by comparing it with empirical distributions.^[39]^ The assessment for the presence of statistical heterogeneity in the entire network will be based on the bias of magnitude of the heterogeneity variance parameter (*τ*^2^) estimated from NMA models using R software (Lucent Technologies, New Jersey, USA).^[39]^ We will also estimate a total *I*^2^ value and predictive intervals for heterogeneity in the network.^[39]^

##### 3.3.6.2 Assessment of transitivity and similarity

We will assess the assumption of transitivity and similarity based on the distribution of clinical and methodological variables that can act as effect modifiers across non-pharmacological intervention comparisons.^[41]^ On the other hand, we will assume that non-pharmacological intervention effects are transitive in this NMA because we will only focus on non-pharmacological interventions, and we will investigate transitivity and similarity based on characteristics of patients, follow-up time and clinical outcomes, clinical characteristics (such as intervention frequency, intervention durations, period of intervention, and severity of depressive symptoms at baseline), as well as according to methodological characteristics, such as study quality.^[39]^

##### 3.3.6.3 Assessment of inconsistency

We will test inconsistency using the node-splitting model.^[42]^ After the data have been scrutinized, possible sources of inconsistency within the clinical and methodological variables will be investigated. The consistency will be evaluated with the *I*^2^ statistics, with an *I*^2^ < 25% indicating mild inconsistency, and 25% < *I*^2^ < 50% showing moderate inconsistency, and an *I*^2^ > 50% representing severe inconsistency.^[39,43]^ If the inconsistency is identified, subgroup analysis, sensitivity analysis, and multiple meta-regressions will be performed.

#### 3.3.7 Publication bias and small-study effects

Potential publication bias will be tested by the visually inspecting the contour-enhanced funnel plots method, the Begg and Mazumdar test and the Egger regression asymmetry test if the number of included studies is larger than 10.^[38,39,44]^ Meta-regression procedures using sample size and effect estimates will be performed to detect the small-study effect.^[38,39]^

#### 3.3.8 Subgroup analysis and sensitivity analysis

When there had been a sufficient studies available, in order to investigate possible the sources of heterogeneity or inconsistency among the results of studies, the subgroup analysis on primary and secondary outcomes will be performed^[38,39]^ as following characteristics: for example, age group, sex ratio, the severity of depressive disorder at baseline, the non-pharmacological intervention duration, injury severity of TBI disease history, time post-injury (acute vs long-term), comorbid general psychiatric disorders, risk of bias, and sample size. Meanwhile, the network meta-regression meta-analysis will be conducted to explore the possible sources of heterogeneity. To verify the robustness of study conclusions, we will perform the sensitivity analysis of outcomes according to methodological quality, study quality, sample size, effect of missing data as well as the analysis methods.^[38,39]^

#### 3.3.9 GRADE quality assessment

Two independent reviewers (MMX and YG) will utilize the Grading of Recommendations Assessment, Development and Evaluation (GRADE) framework to assess the quality of evidence concerning main outcomes and recommendation strength of related interventions.^[45]^ Confidence in network estimates will be divided into 4 levels: high, moderate, low or very low, according to study limitations, inconsistency, indirectness, imprecision, and publication bias of include study.^[45]^

## 4 Ethics and dissemination

This protocol of systematic review and NMA does not require ethics approval and informed consent of patients due to data used on the existing literature. And the final results of this study will be disseminated through conference presentations and publications in peer-reviewed scientific journal.

## 5 Discussion

Depressive disorder appears to be one of most commonly reported disabling psychiatric complication that occurs after TBI.^[9–12]^ Given the negative impact of depressive disorder after TBI on individuals and their social environment, the application of an effective treatment strategy is of considerable importance. Non-pharmacological interventions are popular among patients with depressive disorder. The effectiveness and safety of non-pharmacological interventions to treat depressive disorder has been demonstrated in several reviews,^[46–60]^ but to date there have been very few investigations focusing on non-pharmacological interventions for the treatment of depressive disorder after TBI. In recent years, few studies have reviewed the effective of the individual non-pharmacological intervention for depressive disorder after TBI.^[7,17,30]^ However, the relative therapeutic effect differences among various non-pharmacological intervention are still uncertain. To challenge this open question, this systematic review which aims to quantitatively synthesize the available evidence around the treatment of different non-pharmacological intervention for depressive disorder after TBI. And then the findings of this review will give us a relative ranking of efficacy, effectiveness, safety, acceptability, and tolerability of non-pharmacological interventions while considering both the acute and long-term treatments.

Several strengths should be pay attention in our proposed systematic review. First, we will explore all currently available non-pharmacological options for depressive disorder after TBI reported among eligible high-quality trials. Second, we will update a wide range of search to present date that will include published work in the most comprehensive databases, as well as unpublished work. Third, we will use the GRADE approach to evaluate the quality of evidence supporting intervention effectiveness and safety. In addition, some limitations we must also recognize as follow. First, locating the primary outcome and to run a broad search for them in this systematic review, which may introduce bias to the results since treatment session and measure scale may be various across studies. Second, it is difficult to present single or double-blind trial measures during non-pharmacological intervention, besides, diverse styles of non-pharmacological intervention, characteristics of TBI patients, degree of depressive disorder severity, and study quality may cause statistical heterogeneity. As far as we know, the results of this systematic review will be of interest to a broad audience: practice guideline developers, psychiatrist, neurologist, policymakers and researchers, because it could be used to give clinical recommendations for individual with depressive disorder after TBI, and will also identify gaps in knowledge that could be the subject of future research.

## Acknowledgments

The authors would like to acknowledge Dr Ying Li from School of Acupuncture-Moxibustion and Tuina/The Third Affiliated Hospital, Chengdu University of Traditional Chinese Medicine, and Dr Yimin Zhang from Teaching and Research Section of Acupuncture, School of Traditional Chinese Medicine, Jinan University for providing valuable suggestions to conduct this overview.

## Author contributions

**Conceptualization:** Mingmin Xu, Yu Guo, Yulong Wei.

**Data curation:** Mingmin Xu, Yu Guo, Lu Wang.

**Formal analysis:** Mingmin Xu, Yu Guo, Yulong Wei.

**Investigation:** Mingmin Xu, Lu Wang, Xiumei Feng.

**Methodology:** Mingmin Xu, Yu Guo, Yulong Wei.

**Project administration:** Mingmin Xu, Yu Guo, Yulong Wei.

**Supervision:** Mingmin Xu, Yu Guo, Yulong Wei.

**Validation:** Mingmin Xu, Yue Chen, Jian Yan.

**Visualization:** Mingmin Xu, Yu Guo, Yulong Wei, Lu Wang, Xiumei Feng, Yue Chen, Jian Yan.

**Writing – original draft:** Mingmin Xu, Yu Guo.

**Writing – review & editing:** Mingmin Xu, Yu Guo.
